# Autophagy and mitophagy-related extracellular mitochondrial dysfunction of cerebrospinal fluid cells in patients with hemorrhagic moyamoya disease

**DOI:** 10.1038/s41598-023-40747-9

**Published:** 2023-08-23

**Authors:** Dong Hyuk Youn, Nayoung Kim, Aran Lee, Sung Woo Han, Jong-Tae Kim, Eun Pyo Hong, Harry Jung, Myeong Seon Jeong, Sung Min Cho, Jin Pyeong Jeon, First Korean Stroke Genetics Association Research (The FirstKSGAR) study, In Bok Chang, Seung Hun Sheen, Jong Kook Rhim, Keunsoo Kang, Jun Hyong Ahn, Hong Jun Jeon, Sungyoung Lee, Chan Jong Yoo, Dong Keun Hyun, Jeong Jin Park, Seungwon Kwon, Ian Galea, Ben Gaastra

**Affiliations:** 1 Institute of New Frontier Research, Hallym University College of Medicine, Chuncheon, Korea; 2 Chuncheon Center, Korea Basic Science Institute, Chuncheon, Korea; 3 Department of Neurosurgery, Yonsei University Wonju College of Medicine, Wonju, Korea; 4 Department of Neurosurgery, Hallym University College of Medicine, 77 Sakju-ro, Chuncheon, 24253 Korea; 5 Hallym University College of Medicine, Chuncheon, Korea; 6 CHA University College of Medicine, Pocheon, Korea; 7 Jeju National University College of Medicine, Jeju, Korea; 8 Dankook University College of Science & Technology, Yongin, Korea; 9 Kangwon National University College of Medicine, Chuncheon, Korea; 10 Kangdong Sacred Heart Hospital, Seoul, Korea; 11 Seoul National University Hospital, Seoul, Korea; 12 Gachon University College of Medicine, Incheon, Korea; 13 Inha University College of Medicine, Incheon, Korea; 14 Konkuk University Medical Center, Seoul, Korea; 15 Kyung Hee university College of Medicine, Seoul, Korea; 16 University of Southampton, Southampton, UK

**Keywords:** Neuroscience, Neurology, Risk factors

## Abstract

We aimed to investigate whether mitochondrial dysfunction in extracellular cerebrospinal fluid (CSF), which is associated with autophagy and mitophagy, might be involved in neurological outcomes in adult patients with hemorrhagic moyamoya disease (MMD) whose pathogenesis related to poor outcomes is not well-known. CSF samples were collected from 43 adult MMD patients and analyzed according to outcomes at 3 months. Fluorescence-activated cell sorter analysis (FACS) and the JC-1 red/green ratio were used to assess mitochondrial cells and intact mitochondrial membrane potential (MMP). We performed quantitative real-time polymerase chain reaction and Western blotting analyses of autophagy and mitophagy-related markers, including HIF1α, ATG5, pBECN1, BECN1, BAX, BNIP3L, DAPK1, and PINK1. Finally, FACS analysis with specific fluorescence-conjugated antibodies was performed to evaluate the potential cellular origin of CSF mitochondrial cells. Twenty-seven females (62.8%) with a mean age of 47.4 ± 9.7 years were included in the study. Among 43 patients with hemorrhagic MMD, 23 (53.5%) had poor outcomes. The difference in MMP was evident between the two groups (2.4 ± 0.2 in patients with poor outcome vs. 3.5 ± 0.4 in patients with good outcome; p = 0.02). A significantly higher expression (2^–ΔCt^) of HIF1α, ATG5, DAPK1 followed by BAX and BNIP3L mRNA and protein was also observed in poor-outcome patients compared to those with good outcomes. Higher percentage of vWF-positive mitochondria, suggesting endothelial cell origins, was observed in patients with good outcome compared with those with poor outcome (25.0 ± 1.4% in patients with good outcome vs. 17.5 ± 1.5% in those with poor outcome; p < 0.01). We observed the association between increased mitochondrial dysfunction concomitant with autophagy and mitophagy in CSF cells and neurological outcomes in adult patients with hemorrhagic MMD. Further prospective multicenter studies are needed to determine whether it has a diagnostic value for risk prediction.

## Introduction

Moyamoya disease (MMD) refers to the progressive bilateral occlusion of the internal carotid artery or proximal middle cerebral arteries with unknown etiology. Characteristically, it occurs more frequently in two specific age groups of young children (5 to 10 years of age) and adults (25 to 49 years of age), and it is expressed more in women than in men. MMD is known to be more prevalent in Northeast Asians than in Westerners. The prevalence of MMD in Northeast Asians has been reported to range from 3.16 to 16.1 per 100,000^1^. Also, the incidence rate of adult MMD was increased to 1.74 between 2010 and 2011 based on values reported between 2000 and 2001^2,3^. Although the etiology of MMD is not yet known, it is generally accepted that MMD is accompanied by genetic mutations involving the 17q25 regions of the ring finger protein 213 (RNF 213)^1^. The clinical symptoms of MMD range from asymptomatic to transient ischemic attacks (TIA), cerebral infarction, or intracerebral hemorrhage (ICH)^3^. Adult MMD presents with hemorrhage more frequently compared to pediatric MMD. Among the various types of ICH, intraventricular hemorrhage (IVH) is the most common, approximately 71–82% in hemorrhagic MMD^4–6^. The main research interest so far has been whether or not to reduce the possibility of rebleeding through bypass surgery for adult patients with hemorrhagic MMD^7^, and what radiologic features are associated with bleeding in MMD patients. However, few studies have addressed the patho-mechanism of the aggravation of brain damage that occurs from ICH in adult patients with MMD.

Mitochondria play an important role in generating electrochemical gradients and synaptic transmission for maintaining central nervous system (CNS) system homeostasis^8^. Mitochondrial dysfunction and the subsequent disruptions in metabolic homeostasis cause CNS neuroinflammation and cell death^9^. The mitochondria of endothelial progenitor cells derived from patients with MMD showed morphological disruption with decreased oxygen consumption rates and increased intracellular calcium concentrations^10^. Also, RNF213 was upregulated by mitochondrial dysfunction and inflammatory responses^11,12^. Interestingly, extracellular mitochondria were detected in the cerebrospinal fluid (CSF)^13^. Mitochondria can be transferred via tunneling nanotubes, extracellular vesicles or exosomes under various pathological conditions^9^. Mitochondria exist in various forms in the extracellular space such as active, inactive, malfunctioning or cell-free-circulating mitochondrial DNA^13^. Accordingly, mitochondrial damage or conditions in the CSF may act as a biomarker of disease pathogenesis and outcomes^13^. In particular, it is difficult to obtain affected internal carotid arteries surgically in MMD. Therefore, mitochondrial analysis of the extracellular CSF may offer insights into MMD pathogenesis and predict patient outcomes as in other vascular diseases^14–17^.

Previously, we analyzed the mechanisms associated with mitochondrial dysfunctions and poor outcomes in patients with subarachnoid hemorrhage (SAH), focusing on autophagy and mitophagy in CSF cells. Specifically, increased mitochondrial dysfunction associated with autophagy and mitophagy might be involved in delayed cerebral ischemia (DCI) pathogenesis after SAH^18^. Based on this research experience, we hypothesized that mitochondrial dysfunction associated with autophagy and mitophagy in CSF cells would be associated with neurological outcomes in adult patients with hemorrhagic MMD whose pathogenesis related to poor outcomes is not well known. To confirm our hypothesis, we collected the extracellular CSF cells of adult patients with hemorrhagic MMD and investigated the association with neurologic outcomes by analyzing the expression of autophagy and mitophagy-related markers in the cells.

## Methods

### Study population

**Figure 1:**
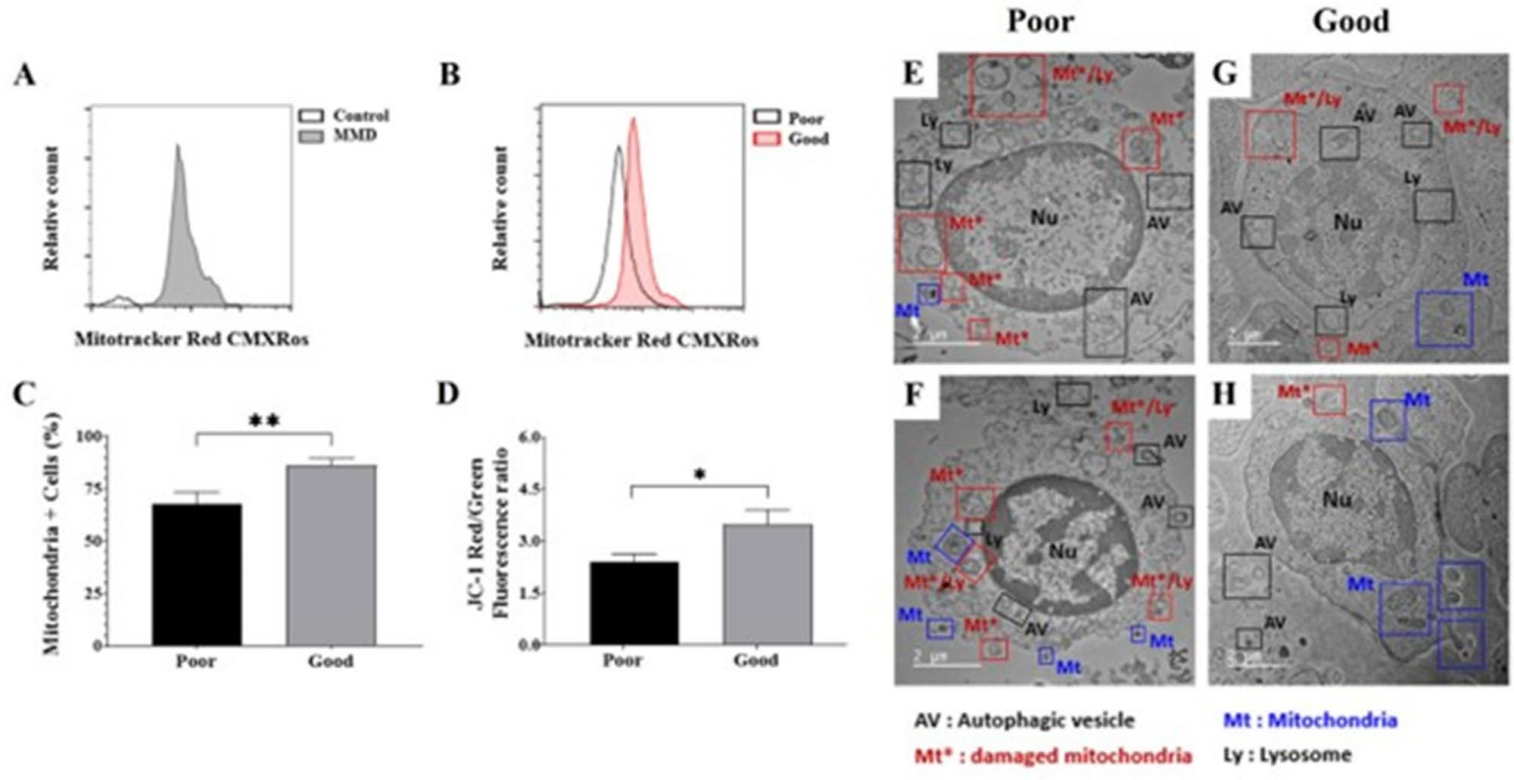
Comparison of flow cytometry analysis results CSF cells between adult patients with hemorrhagic moyamoya disease (MMD) and control subjects (**A**) and neurological outcome in MMD patients (**B**) using MitoTracker Red CMXRos (200 nM). Differences in the number of mitochondrial cells (**C**) and mitochondrial membrane potential using the JC-1 red/green ratio (**D**) according to outcomes. Representative images of transmission electron microscopy illustrating mitochondrial dysfunction in the CSF cells of hemorrhagic MMD patients with good and poor outcomes. Damaged mitochondria and autophagic vacuoles with matrix swelling and collapsed cristae were observed more frequently in poor-outcome patients (**E**,**F**) than in good-outcome patients (**G**,**H**). *Nu* nucleus, *Mt* mitochondria, *Ly* lysosome, *AV* autophagic vesicle, Mt* damaged mitochondria, Mt*/*Ly* fusion of autophagic vacuoles with damaged mitochondria. Scale bar = 2 μm.

The study participants were derived from an ongoing prospective multicenter stroke database from March 2016 to February 2022 (http://1ksgh.org/)^18–21^. MMD diagnosis was performed by referring to a previous report^22^. More specifically, adult patients (≥ 18 years) with typical MMD angiographic features unilaterally or bilaterally were initially enrolled. Then, the exclusion criteria were applied based on risk factors, such as atherosclerosis, congenital anomaly, and prothrombotic past medical history, according to a previous study^22^. CSF samples from MMD patients with intracranial malignancy, infectious meningitis, end-stage hepatic or renal disease, and pregnancy were also excluded^16^. The treatment protocol for patients with hemorrhagic MMD was as follows: (1) cerebral angiography was performed to confirm whether there was an aneurysm that could cause bleeding and if so, surgery was performed; and (2) if increased intracranial pressure (IICP) was observed, extraventricular drainage (EVD) or craniotomy and hematoma removal was performed as needed. In the intensive care unit, lumbar CSF drainage was usually performed for 5 to 7 days. CSF samples within 72 h were collected and stored for analysis. CSF samples from two patients with idiopathic hydrocephalus were used as the control group (Fig. 1A). Two investigators reviewed the medical records (e.g. age, gender, underlying diseases, smoking, familial history of MMD, and prior MMD-related symptoms) and radiological findings (e.g. hemorrhagic pattern, bleeding focus, and Suzuki stage)^5^. A good neurologic outcome was defined as a modified Rankin scale score (mRS) of 0 to 3 at 3 months after first bleeding^5^. Specific mRS scores associated with good outcomes include: 0, no symptoms; 1, no significant disability; 2, slight disability, but able to look after own affairs without assistance; and 3, moderate disability that requires some help, but able to walk unassisted. This study was approved by the Institutional Review Board (No. 2017-9, 2018-6, and 2019-6) of the of the Hallym University-Chuncheon Sacred Hospital. All methods were performed in accordance with the Chuncheon Sacred Hospital guidelines and regulations. Informed consent was obtained from the patients or their relatives. Study was performed according to the STROBE guidelines.

### Fluorescence-activated cell sorter analysis

Fluorescence-activated cell sorter analysis (FACS) was performed to identify extracellular mitochondria in the CSF samples (BD FACSCalibur, Becton–Dickinson, San Jose, CA, USA), according to our previous reports^17,18^. A 100-µl CSF sample was stained with 200 nM MitoTracker Red CMXRos (Thermo Fisher Scientific, Waltham, MA, USA) for 40 min at room temperature. The potential cellular origin of the extracellular mitochondrial signals was evaluated using the following fluorescence-conjugated antibodies: von Willebrand factor conjugated with fluorescein isothiocyanate (vWF-FITC; Abcam, Cambridge, MA, USA), glutamate aspartate transporter conjugated with allophycocyanin (GLAST-APC, Miltenyl Biotec, Madrid, Spain), CD45-FITC (Miltenyl Biotec), and CD41/61-FITC (Miltenyi Biotec) (Supplementary Table S1). The CSF samples were immunostained with the antibodies for 20 min at room temperature and analyzed by flow cytometry. The data were analyzed using Flow Jo software (v 10.7.1 Ashland, OR, USA)^17,18^.

### Mitochondrial membrane potential

MMP was measured in the CSF cells using a MitoProbe JC-1 assay kit (Thermo Fisher Scientific)^16–18,23^. JC-1 monomers emit green fluorescence (excitation/emission, 485/516). When they enter the mitochondria, JC-1 forms complexes known as J-aggregates, emitting high red fluorescence intensity from red aggregates (excitation/emission, 570/595). The JC-1 ratio of red to green fluorescence is generally used as a parameter to trace MMP. In brief, human CSF samples (100 µL) were incubated with 1 µL of 1 µM in dimethyl sulfoxide of JC-1 for 25 min at 37 °C in the dark. The fluorescence intensity was measured using GloMax Explorer (Promega, WI, USA). The MMP ratio was estimated by dividing the red fluorescence value by the green value.

### Field emission transmission electron microscopy

CSF samples were centrifuged (2500 rpm, 5 min, room temperature) and examined by field emission electron microscopy (FE-TEM) by referring to previous protocols^17,18^. FE-TEM was used to identify autophagic vacuoles and morphological changes in mitochondria in the CSF cells^18^. The pellets were fixed overnight in 2% glutaraldehyde in cacodylate buffer (0.1 M sodium cacodylate, 2 mM MgCl2, pH 7.4) at 4 °C. After washing three times with cacodylate buffer at 4 °C, the samples were post-fixed in 2% osmium tetroxide for 1 h at 4 °C. The samples were rinsed with deionized water and dehydrated in a graded ethanol series of 50% to 100% for 20 min at each step. The samples were incubated with progressively more concentrated propylene oxide dissolved in ethanol, followed by infiltration with increasing concentrations of Eponate 812 resin. The samples were dried in a 65 °C oven overnight and sectioned using an ultramicrotome. The sections were observed with an FE-TEM unit (JEM-2100F, JEOL) at the Korean Basic Science Institute (Chuncheon, Korea)^17,18^.

### Quantitative real-time reverse transcription analysis

We measured the mRNA expression of autophagy and mitophagy-related markers, including hypoxia-inducible-factor 1α (HIF1α), autophagy-related gene 5 (ATG5), pBECN1, BECN1 (ATG6), Bcl-1 antagonist X (BAX)/Bcl-2 ratio, BCL2 interacting protein 3 like (BNIP3L), death-associated protein kinase (DAPK)-1, and phosphatase and tensin homolog (PTEN)-induced kinase 1 (PINK1)^17,18^. Total RNA was isolated from the CSF cells using the easy-BLUE RNA extraction kit (iNtRON Biotechnology, Korea) according to the manufacturer’s instructions. cDNA was synthesized from 1 μg of RNA using the Maxime RT PreMix Kit (iNtRON Biotechnology, Korea). mRNA expression was measured by quantitative real-time reverse transcription (qRT-PCR) analysis using 2 × Rotor-Gene SYBR Green qPCR Master Mix (Qiagen, Carlsbad, CA, USA) in the Rotor-Gene Q (Qiagen). The PCR primers used in this study are presented in Supplementary Table S2.

### Western blotting analysis

Western blotting was performed as in our previous studies^17,18^. CSF cells were lysed with RIPA buffer (50 mM Tris-base, 10 mM EDTA, 150 nM NaCl, 0.1% sodium dodecyl sulfate (SDS), 1% Triton X-100, 1% sodium deoxycholate, 1 mM phenylmethylsulfonyl fluoride (PMSF). Protein lysates of the supernatant were quantified using the BCA protein assay kit (Thermo Scientific). Equal amounts of protein were separated by 10 and 15% SDS–polyacrylamide gel electrophoresis (SDS-PAGE) and transferred to polyvinylidene fluoride (PVDF) membranes (Bio-Rad, USA). After blocking the PVDF membranes with a double blocker (phosphate-buffered saline buffer-based BDP) for 1 h, the transferred PVDF membranes were incubated with the primary antibodies overnight at 4 °C. After washing in Tris-buffered saline containing 0.1% Tween-20 (TBS-T buffer), the membranes were incubated with horseradish peroxidase-conjugated secondary antibodies for 1 h at room temperature and developed using an enhanced chemiluminescence (ECL) Western blotting substrate kit (Thermo Fisher Scientific). The antibodies used in this study are presented in Supplementary Table S3.

### Statistical analysis

Continuous variables are presented as the mean and ± standard deviation (SD). The chi-squared or Student’s t test was used to compare differences in the variables. Western blot reactions were quantified by estimating the optical density relative to that of actin used as the reference value.

The degree of qRT-PCR and western blots were described as the mean ± standard error of the mean (SEM). Statistical analysis was performed with SPSS V.21 (SPSS, IL, USA) and GraphPad Prism software (v.6.01; GraphPad Software Inc., San Diego, CA, USA). Statistical significance was indicated at p-values of < 0.05. P-values of less than of < 0.05 and 0.01 are represented by * and **, respectively.

## Results

### Clinical characteristics of the enrolled patients

A total of 43 adult patients with hemorrhagic MMD, including 27 females (62.8%), with a mean age of 47.4 ± 9.7 years, were enrolled for the analysis after exclusions (Supplementary Fig. S1). Eight (18.6%) patients had a familial history of MMD, and 14 (32.6%) had prior ischemic symptoms before bleeding. The number of unilateral MMD and posterior cerebral artery (PCA) involvements was 9 (20.9%) and 9 (20.9%), respectively. Among the various ICH types, IVH (n = 21, 48.8%), ICH with IVH (n = 10, 23.3%), and ICH (n = 9, 20.9%) occurred most frequently. Twenty-three patients (53.5%) underwent conservative treatment, and 20 patients underwent surgical treatment. EVD was performed in 13 patients, and craniotomy and hematoma removal in 7 patients, among which 2 (2/7) patients had coil embolization prior to surgery to treat an aneurysm thought to be the cause of the bleeding (Table 1).

**Table 1:** Clinical and radiological characteristics of the enrolled patients with adult hemorrhagic moyamoya disease.

Variables	Number of patients (%)
Clinical characteristics	
Female	27 (62.8%)
Age, years	47.4 ± 9.7
Hypertension	5 (11.6%)
Diabetes mellitus	4 (9.3%)
Hyperlipidemia	4 (9.3%)
Smoking	6 (14.0%)
Familial history	8 (18.6%)
Prior ischemic symptoms	14 (32.6%)
Radiologic findings	
Unilateral MMD	9 (20.9%)
Suzuki grade	3.9 ± 1.2
PCA involvement	9 (20.9%)
Type of hemorrhage	
IVH	21 (48.8%)
ICH	9 (20.9%)
ICH with IVH	10 (23.3%)
SAH	3 (7.0%)
Treatment at first bleeding	
Conservative treatment	23 (53.5%)
Extraventricular drainage	13 (30.2%)
Hematoma removal	7 (16.3%)

*PCA* posterior cerebral artery, *IVH* intraventricular hemorrhage, *ICH* intracranial hemorrhage, *SAH* subarachnoid hemorrhage.

Three months after ictus, 23 (53.5%) patients had poor neurological outcomes. The presence of IVH was observed more in patients with poor outcomes (n = 18, 78.3%) than in those with good outcomes (n = 13, 65.0%), but the difference was not statistically significant (p = 0.334). Surgical treatment was also observed more in patients with poor outcomes (n = 12, 52.2%), but the difference was not statistically significant (p = 0.425). Overall, the analysis of clinical and radiological variables among adult patients with hemorrhagic MMD showed that no variables were closely related to patient outcomes at 3 months (Table 2).

**Table 2:** Comparison of clinical and radiologic findings, treatment types, and autophagy and mitophagy-related mRNA expression according to patient outcomes.

Variables	Good outcome (n = 20)	Poor outcome (n = 23)	p-value
Clinical findings			
Female	14 (70.0%)	13 (56.5%)	0.853
Age, years	46.5 ± 11.0	48.3 ± 8.5	0.554
Hypertension	2 (10.0%)	3 (13.0%)	0.756
Diabetes mellitus	2 (10.0%)	2 (8.7%)	0.883
Hyperlipidemia	1 (5.0%)	3 (13.0%)	0.385
Smoking	2 (10.0%)	4 (17.4%)	0.485
Familial history	3 (15.0%)	5 (21.7%)	0.571
Prior ischemic symptoms	6 (30.0%)	8 (34.8%)	0.739
Radiologic findings			
Unilateral MMD	4 (20.0%)	5 (21.7%)	0.889
Suzuki grade	3.7 ± 1.1	4.1 ± 1.3	0.302
Presence of IVH	13 (65.0%)	18 (78.3%)	0.334
PCA involvement	4 (20.0%)	5 (21.7%)	0.889
Treatment at first bleeding			
Conservative treatment	12 (60.0%)	11 (47.8%)	0.425
Surgical treatment	8 (40.0%)	12 (52.2%)	
Autophagy and mitophagy markers*			
HIF1α	0.359 ± 0.069	0.581 ± 0.078	0.041
ATG5	0.024 ± 0.005	0.059 ± 0.014	0.036
BECN1	0.180 ± 0.038	0.141 ± 0.036	0.462
BAX	0.166 ± 0.031	0.355 ± 0.083	0.047
BNIP3L	0.059 ± 0.017	0.147 ± 0.031	0.022
DAPK1	0.012 ± 0.002	0.034 ± 0.010	0.042
PINK1	0.089 ± 0.027	0.061 ± 0.017	0.388

*The mRNA (2^–ΔCt^) level of autophagy and mitophagy markers are presented as mean with SEM.

*ATG5* indicates autophagy-related gene 5, *BAX* Bcl-1 antagonist X, *BNIP3L*, *BCL2* interacting protein 3-like, *DAPK*-1 death-associated protein kinase-1, *HIF1α* hypoxia-inducible-factor 1A, *IVH* intraventricular hemorrhage, *LC3B* light chain 3B, *MMD* moyamoya disease, *PCA* posterior cerebral artery, *PINK1*, *PTEN*-induced kinase 1.

### FACS, MMP, and TEM

FACS analysis revealed that patients with hemorrhagic MMD exhibited more extracellular mitochondria in CSF cells than control subjects (Fig. 1A). Patients with good outcomes had significantly higher numbers of extracellular mitochondria in the CSF than patients with poor outcomes (Fig. 1B and C, and Supplementary Fig. S3). Poor-outcome patients (2.4 ± 0.2) showed lower MMP levels according to the JC-1 red/green ratio compared to good-outcome patients (3.5 ± 0.4; p = 0.02) (Fig. 1D).

FE-TEM images of CSF cells were compared according to neurological outcomes (Fig. 1E–H). Poor-outcome patients had more damaged mitochondria, with autophagic vacuoles containing damaged mitochondria in the CSF, than good-outcome patients. Also, damaged mitochondria had matrix swelling and collapsed cristae. These findings suggest that mitochondrial dysfunction associated with autophagy and mitophagy in CSF cells after a hemorrhagic event reflects MMD severity, which may be related to the prognosis of adult patients with hemorrhage MMD.

### mRNA and protein expression

**Figure 2:**
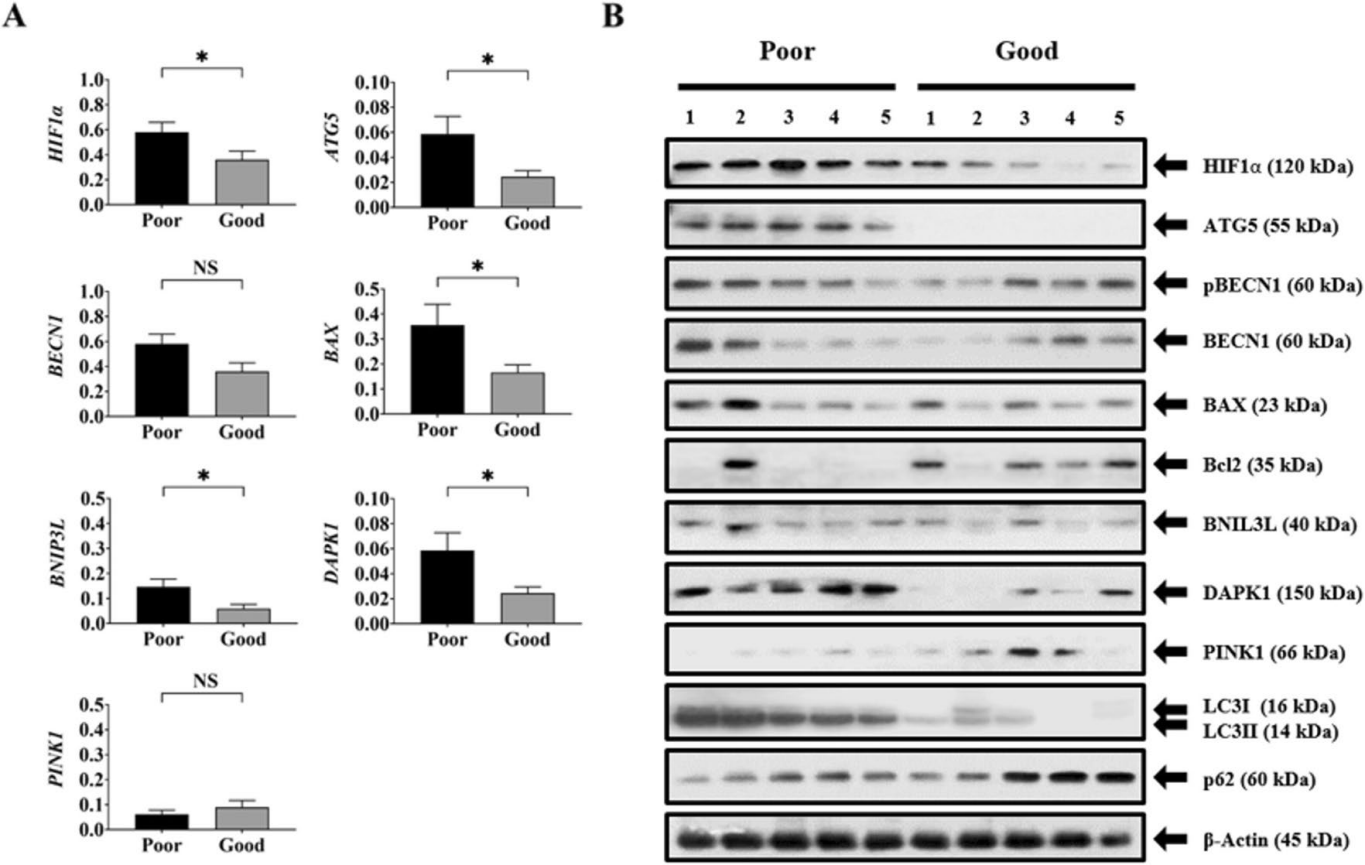
(**A**,**B**) mRNA and protein expression of autophagy and mitophagy-related markers in adult hemorrhagic MMD patients with good and poor outcomes. Actin was used as the loading control. The error bars represent SEM. P-values of less than < 0.05 and 0.01 are represented by * and **, respectively. *NS* not significant.

The mRNA and protein expression of autophagy and mitophagy-related markers is shown in Fig. 2. Compared to good-outcome patients, poor-outcome patients showed significantly higher expression (2^–ΔCt^) of HIF1α (0.359 ± 0.069 vs. 0.581 ± 0.078; p = 0.041), ATG5 (0.024 ± 0.005 vs. 0.059 ± 0.014; p = 0.036), BAX (0.166 ± 0.031 vs. 0.355 ± 0.083; p = 0.047), BNIP3L (0.059 ± 0.017 vs. 0.147 ± 0.031; p = 0.022), and DAPK1 (0.012 ± 0.002 vs. 0.034 ± 0.010; p = 0.042). Other makers, such as BECN1 and PINK1, did not differ significantly between the two groups. Western blotting analyses revealed that poor outcomes patients showed significantly higher expression of HIF1α (0.456 ± 0.045 in good outcome vs. 0.720 ± 0.057 in poor outcome), ATG5 (0.089 ± 0.006 in good outcome vs. 0.532 ± 0.075 in poor outcome), and DAPK1 (0.144 ± 0.070 in good outcome vs 0.468 ± 0.064 in poor outcome) followed by BAX/Bcl-2 compared to those with good outcome (Supplementary Fig. S2).

### Potential cellular origin of mitochondria

**Figure 3:**
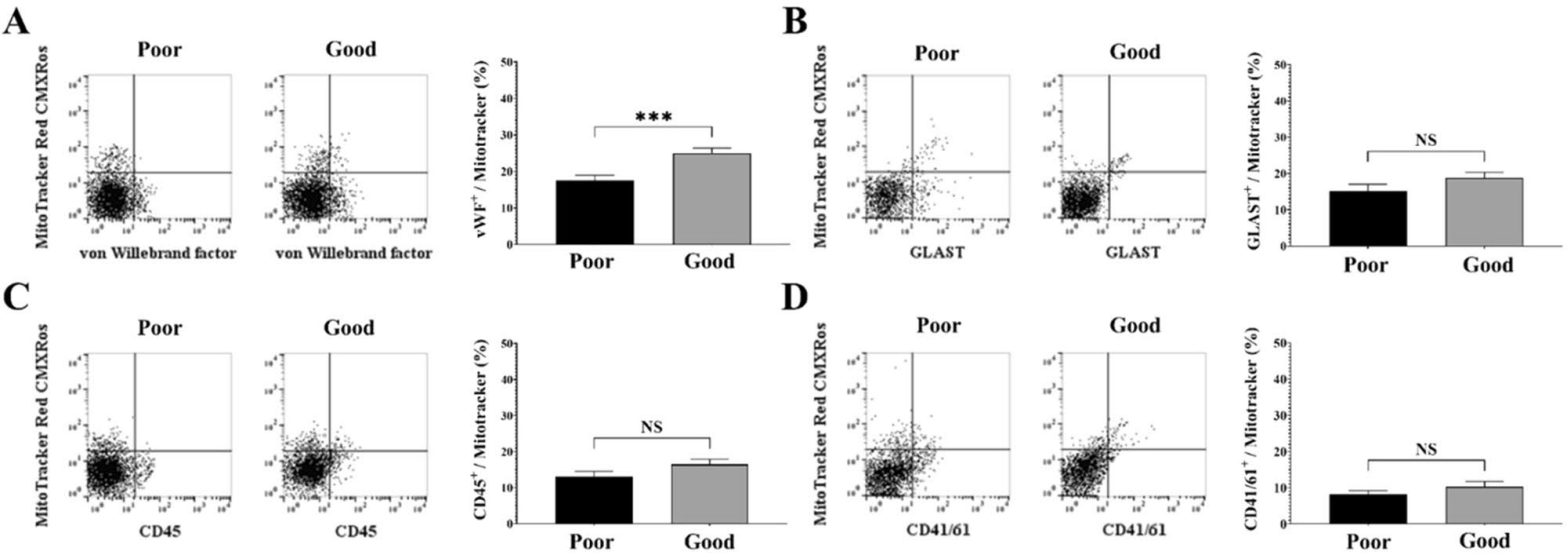
Analysis of the potential origin of extracellular mitochondria in CSF cells in adult hemorrhagic MMD patients according to outcomes. The cell markers used in this fluorescence-activated cell sorter analysis of CSF cells and their possible origin are as follows: Willebrand factor (vWF-FITC; endothelial) (**A**), glutamate-aspartate transporter (GLAST-APC; astrocyte) (**B**), cluster of differentiation 45 (CD45-FITC; microglia/macrophage) (**C**), and cluster of differentiation 41/61 (CD41/61-FITC; platelet) (**D**). *NS* not significant.

To evaluate the potential cellular origin of the mitochondria in CSF cells, we performed FACS analysis with specific fluorescence-conjugated antibodies^18^. Each antibody conjugate assessed the following cellular origins: vWF (endothelial cells), GLAST (astrocyte), CD41/61 (platelet), and CD45 (microglia/macrophage)^16^. Good-outcome patients had significantly higher percentages of vWF-positive mitochondria (25.0 ± 1.4%) than poor-outcome patients (17.5 ± 1.5%; p < 0.01). Other variables, such as GLAST (18.8 ± 1.5% in good outcome vs. 15.2 ± 1.8% in poor outcome; p = 0.14), CD45 (16.5 ± 1.4% in good outcome vs. 13.0 ± 1.5% in poor outcome; p = 0.11), and CD41/61 (10.3 ± 1.4% in good outcome vs. 8.2 ± 1.1% in poor outcome; p = 0.22) did not differ significantly according to outcomes (Fig. 3).

## Discussion

This study evaluated autophagy and mitophagy markers in CSF cells to reveal the underlying mechanism of neurological deterioration in adult patients with hemorrhagic MMD by focusing on mitochondrial dysfunction. Our study, for the first time, showed that mitochondria in CSF from adult patients with hemorrhagic MMD differed according to outcomes. First, good outcome patients had more CSF mitochondria with high MMP. Second, increased mitochondrial dysfunction associated with autophagy and mitophagy in CSF cells may contribute to neurological deterioration. Among the markers, the expression of HIF1α, ATG5, and DAPK-1 varied most significantly according to outcomes. Third, a higher percentage of mitochondria potentially originating from endothelial cells were observed more frequently in adult hemorrhagic MMD patients with good outcomes than those with poor outcomes.

MMD has been regarded as a model of chronic hypoperfusion. MCA specimens from MMD patients were shown to exhibit thicker intima walls with an increased expression of HIF1α compared to controls^24^. HIF1α regulates oxygen homeostasis. The rate of hydroxylation decreases under ischemic conditions due to mitochondrial reactive oxygen species (ROS) production or inadequate O_2_ substrate^25^. Zhang et al.^25^ reported that mitophagy was an adaptive metabolic response to hypoxia via the HIF1α-dependent pathway. In particular, HIF1α-mediated responses are beneficial by reducing ROS levels and cell death by acute cellular exposure to ischemic conditions. Li et al.^26^ also reported that mitochondrial HIF1α exerted protection against oxidative stress. When ICH occurs in chronic cerebral ischemia due to MMD, an acute cerebral ischemia condition develops due to the sudden increased intracranial pressure (IICP). Accordingly, we think that increased HIF1α in adult hemorrhagic MMD patients with poor outcomes may be a compensatory process to protect against oxidative stress that targets mitochondria. Autophagy and mitophagy regulate inflammatory responses in the CNS. In particular, the nucleotide-binding oligomerization domain-like receptor protein 3 (NLRP3) inflammasome is activated immediately after ICH. Cao et al.^27^ showed that melatonin increased the expression of autophagy markers ATG5 and LC3-II/LC3-I and mitophagy markers Parkin and PINK-1, with decreased NLRP3 inflammasome activation following SAH. Conversely, an autophagy inhibitor reversed these melatonin effect on autophagy and mitophagy and the NLRP3 inflammasome. ATG5 plays an important role in both canonical and non-canonical autophagy processes. ATG5 was reported to form a complex by binding with ATG12 and ATG16 and was deposited on the autophagosome membrane after conjugation with LC3 and phosphatidylethanolamine (PE)^28^. ATG5 is also involved in LC3-associated phagocytosis. Through these processes, ATG5 is involved in the fusion of autophagosomes and lysosomes^28^. Our results illustrated higher expression of ATG5 in the CSF cells of poor-outcome patients compared to good-outcome patients. The results suggest that autophagic function was increased to reduce ROS and neuroinflammation in patients with severe hemorrhagic MMD. However, it is still unclear whether increased autophagy with increased ATG5 expression has a protective or detrimental effect in adult patients with hemorrhagic MMD. Under ischemic-reperfusion conditions, ATG5 knockdown decreased brain injury by reducing excessive autophagy-induced ferroptosis^29^. Accordingly, further research on the mechanism of how ATG5 is associated with brain protection and good neurological outcomes in adult MMD patients after ICH development is required.

DAPK1 is a regulator of autophagy and apoptosis and acts as a crucial component in mitochondrial toxin-induced cell death^30,31^. Few studies on the role of DAPK1 in MMD have been conducted compared to neurodegenerative diseases or cancer. DAPK1 knockout mice exhibited decreased inflammation and vascular injury to arterial hypertension-induced aneurysms^32^. Beclin1 also affects the action of DAPK1 on inflammation associated with aneurysm formation via ATP-dependent NLRP3 inflammasomes^32^. DAPK1 was dephosphorylated, and its mRNA expression was activated in vivo and in vitro models of cerebral ischemia, resulting in an increase in the binding of mitochondrially translocated p53 to CypD^33,34^. In addition, DAPK1 activation decreased MMP with subsequent mitochondrial swelling in neuroblastoma cells^18,35^. Tu et al.^36^ reported that DAPK1 directly bound to N-methyl-D-aspartate (NMDA) receptor NR2B subunits at Ser-1303, resulting in increased NR1/NR2B receptor channel conductance and calcium influx in a stroke mouse model. However, future studies should investigate the specific role of DAPK1 in extracellular mitochondria in the CSF cells of adult patients with hemorrhagic MMD.

MMD vessels revealed a marked accumulation of hyaluronan and chondroitin sulfate in the thickened intima. Hyaluronan synthase 2 was also highly expressed in endothelial progenitor cells^37^. Matsuo et al.^37^ reported that an altered peri-endothelial matrix in the MMD vessel might increase endothelial vulnerability to wall shear stress, causing the excessive accumulation of hyaluronan and chondroitin sulfate in the intima of MMD vessels. Interestingly, a higher percentage of vWF-positive mitochondria was observed in adult hemorrhagic MMD patients with good outcomes than in those with poor outcomes in our study. The transfer of endothelial cells into the CSF after ICH development was observed in patients with SAH but not MMD^16–18^. SAH patients had a higher synthesis of endothelial microparticles containing CD105^+^ and CD62e^+^ compared to controls^38^. Youn et al.^18^ reported that DCI patients exhibited a higher percentage of vWF-positive mitochondria in CSF cells than non-DCI patients. However, the difference in vWF-positive mitochondria in CSF cells was not significantly associated with the outcomes of SAH patients^16^. From our study results alone, it is not possible to know how the transfer of mitochondria, possibly originating from endothelial cells, to the CSF space, is linked to good neurological outcomes in adult patients with hemorrhagic MMD. Accordingly, future studies should focus on the mitochondria of vascular endothelial cells as these extracellular mitochondria may reflect metabolic integrity and the degree of brain damage after bleeding in MMD.

RNF213 gene at 17q25 which encodes mysterin represents a major susceptibility gene underlying MMD^39^. In particular, p.R4810K mutation has been associated with advanced MMD vasculature, posterior arterial circulation, and earlier age of onset^12,40^. Wang et al.^40^ reported that p.R4810K was significantly associated with patients with MMD compared with controls [OR 48.1 (95% CI 29.1–79.6)]. The onset age of MMD with the GA and AA genotypes was younger than in those carrying GG genotypes^40^. Also, lower rates of recurrent stroke and better outcomes were observed in MMD associated with GA and AA genotypes compared with GG genotypes^41^. The protein mysterin contains two tandem AAA + ATPase modules, resulting in the formation of a ring-shaped oligomeric complex^42^. Key et al.^11^ showed that genetic ablation of mitochondrial matrix factors including the caseinolytic peptidase P (ClpP) and the transcription factor Tfam potently induced the transcription of RNF213 in various organs, suggesting that mysterin plays a role MMD-like vasculopathies via mitochondrial dysfunction as well as RNA-dependent inflammation^11^. In this study, we did not analyze the association between RNF213 variants and mitochondrial dysfunction, and neurological outcomes. Therefore, further studies involving large patient populations are needed to determine differences in extracellular mitochondrial dysfunction based on R4810K (c.14429G > A, rs112735431) mutations in RNF213.

There were some limitations to this study. First, we enrolled adult MMD patients with hemorrhagic presentation, but not asymptomatic or ischemic presentation. In MMD patients who are asymptomatic or manifest with cerebral ischemia, CSF drainage is not routinely performed. Although we used patients with hydrocephalus as a control group, mitochondrial cells in asymptomatic or ischemic-presenting MMD patients may have other CSF characteristics. Second, we did not conduct an additional mechanistic study to determine how increased mitophagy and autophagy responses are associated with neurologic outcomes in adult patients with hemorrhagic MMD. The inhibition of autophagy contributes to increased cell death, as well as cytotoxicity^43^. Although excessive autophagy and mitophagy in chronic cerebral hypoperfusion can induce brain damage^44^, it is difficult to make in vivo and in vitro models of MMD. Third, which is an extension of the second limitation, general mitochondrial dysfunction occurs in the CSF after cerebral hemorrhage, not specifically in patients with adult MMD presenting with hemorrhage manifestations. Of course, there were conspicuous differences in results between our study and previous study. 18 First, a higher percentage of vWF-positive mitochondria was observed in patients with good outcome compared with those exhibiting poor outcomes in hemorrhagic MMD. By contrast, the number of vWF-positive mitochondria were increased in patients with SAH associated with DCI compared with those without DCI. Second, the expression of HIF1α, ATG5, and DAPK-1 showed the maximum difference based on outcomes involving adult patients with hemorrhagic MMD, whereas in SAH, the differences in expression of DAPK1, BNIP3L, and PINK1 were the most prominent. Nevertheless, mechanistic studies are needed to determine whether extracellular mitochondrial dysfunction is disease-specific or a general phenomenon. Fourth, the difference in the degree of IVH and cellularity (ICH vs. IVH) may affect the statistical significance of our results. A multivariate analysis of clinical and imaging variables as well as mitochondrial markers is essential to determine the role of mitochondrial dysfunction in neurological outcomes after a hemorrhagic event in adult MMD. However, it was impossible with our sample size of 43. Accordingly, further studies involving a large number of patients are needed to confirm our findings. Nevertheless, our study was the first to investigate mitochondrial dysfunction associated with autophagy and mitophagy in the CSF cells of adult MMD patients with hemorrhagic presentations.

In conclusion, our study revealed that extracellular mitochondria in CSF cells exhibited a dynamic nature after hemorrhage in adult MMD patients. Mitochondrial dysfunction, associated with autophagy and mitophagy in the CSF cells, may also be associated with neurological outcomes in adult patients with hemorrhagic MMD. In particular, the autophagy and mitophagy-associated markers of HIF1α, ATG5, and DAPK1 were significantly increased in patients with poor outcomes compared to those with good outcomes. Also, the number of CSF mitochondrial cells, possibly originating from endothelial cells, differed significantly according to outcomes. These findings provide new insights into the pathogenesis of adult MMD patients with hemorrhagic presentations.

### Supplementary Information

Supplementary Information.

## Data Availability

The datasets used in this study are available from the corresponding author on reasonable request.

## Abbreviations

ATG5: Autophagy-related gene 5
BAX: Bcl-1 antagonist X
CSF: Cerebrospinal fluid
CNS: Central nervous system
DAPK: Death-associated protein kinase
DCI: Delayed cerebral ischemia
EVD: Extraventricular drainage
FACS: Fluorescence-activated cell sorter analysis
FE-TEM: Field emission electron microscopy
GLAST-APC: Glutamate aspartate transporter conjugated with allophycocyanin
HIF1α: Hypoxia-inducible-factor 1α
ICH: Intracerebral hemorrhage
IICP: Increased intracranial pressure
MMD: Moyamoya disease
MMP: Mitochondrial membrane potential
mRS: Modified Rankin scale score
PINK1: Phosphatase and tensin homolog-induced kinase 1
PVDF: Polyvinylidene fluoride
qRT-PCR: Quantitative real-time reverse transcription
ROS: Reactive oxygen species
SDS: Sodium dodecyl sulfate
vWF-FITC: Von Willebrand factor conjugated with fluorescein isothiocyanate
