# Supplementary material for: Autophagy and mitophagy-related extracellular mitochondrial dysfunction of cerebrospinal fluid cells in patients with hemorrhagic moyamoya disease
Source: Sci Rep. 2023 Aug 23;13:13753. doi: 10.1038/s41598-023-40747-9 (PMC10447448; doi:10.1038/s41598-023-40747-9)
Supplement: Supplementary file 1 — Supplementary Information. [file 41598_2023_40747_MOESM1_ESM.docx]

**SUPPLEMENTAL DATA**

**Supplemental Figure S1.** Detailed scheme of the study.

**
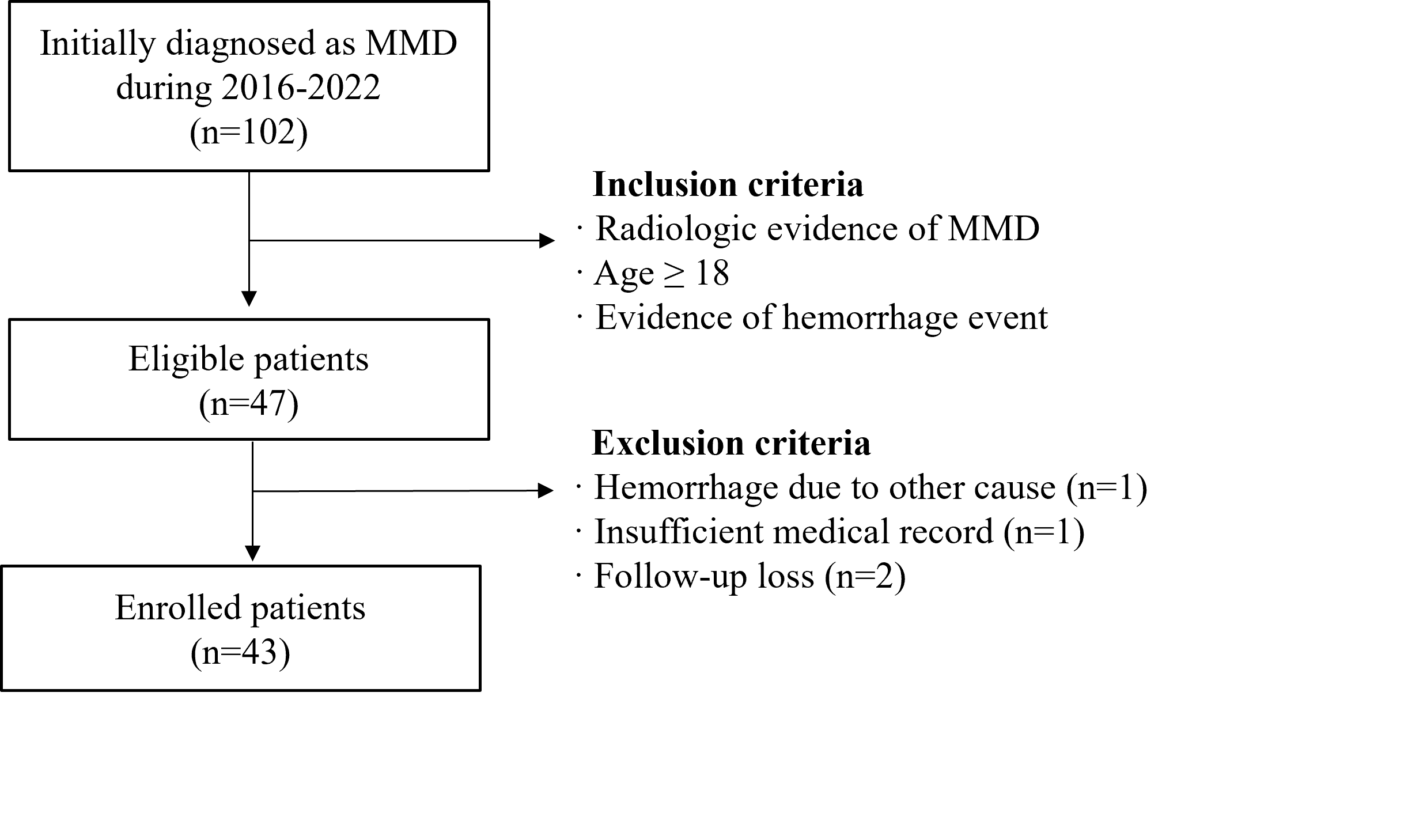
**

**Supplemental Figure S2.** Representative full scanned images of original unprocessed Western blotting (A) and quantitative analysis (B) according to outcomes.


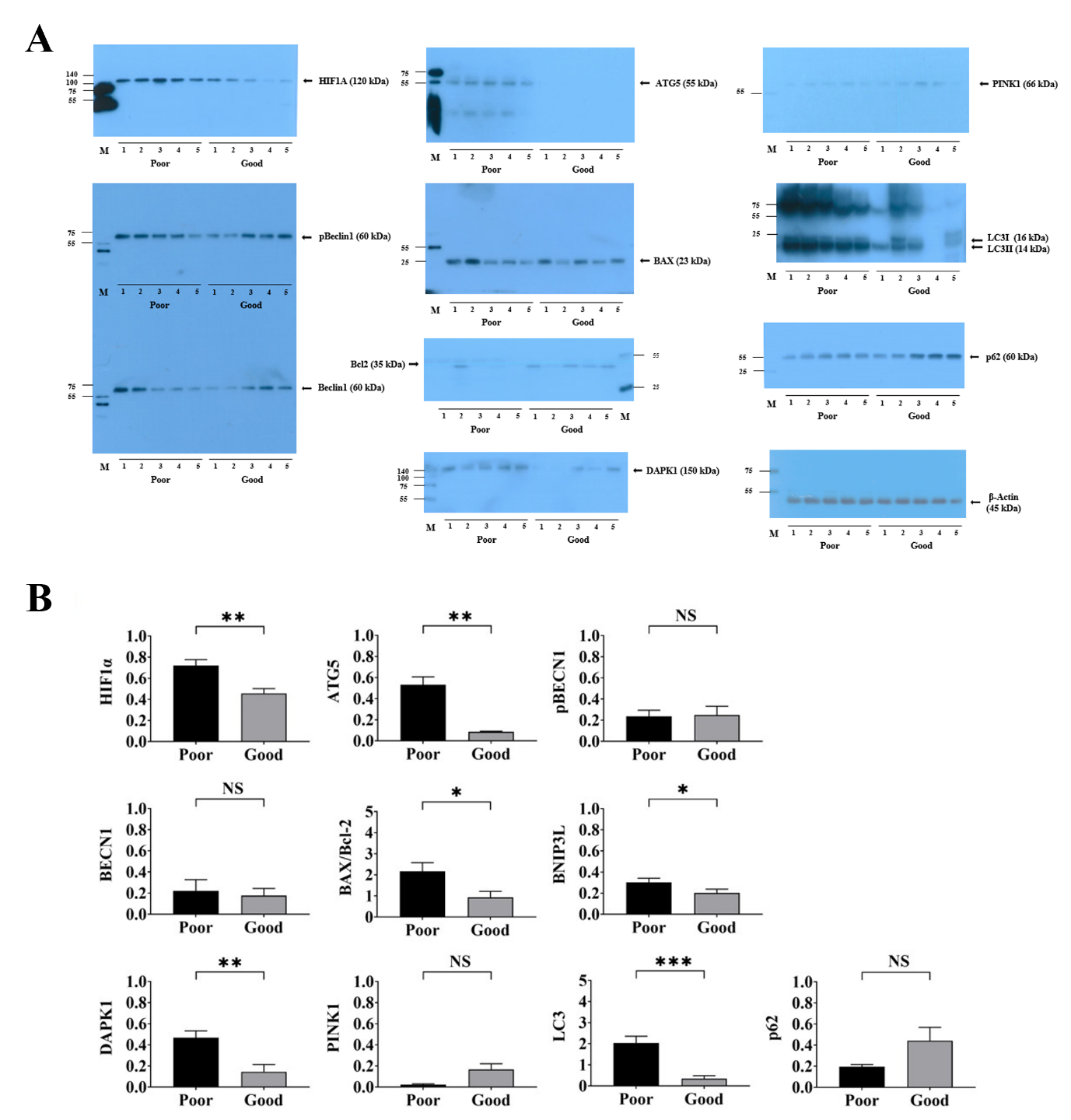


**Supplemental Figure S3.** Representative magnified images acquired via transmission electron microscopy show mitochondrial dysfunction in the CSF cells of patients with poor outcomes in hemorrhagic MMD. Damaged mitochondria and autophagic vacuoles were observed with swollen matrix and collapsed cristae.


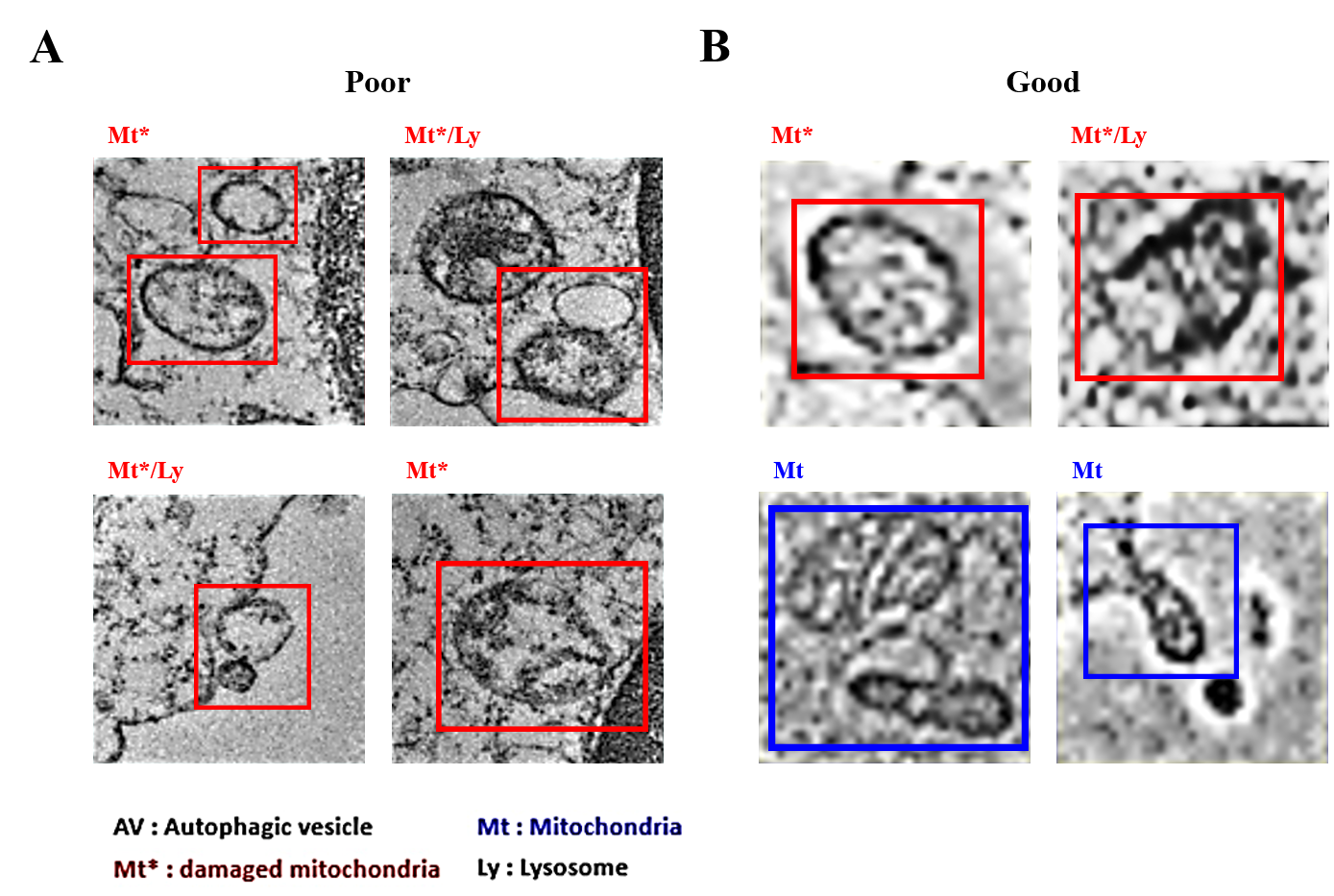


**Supplemental Table S1.** List of the fluorescence-activated cell sorter analysis antibodies used in this study.

| No. | Antibody for FACS | Company | Cat. No. | Clone | Used concentration of  stock solution |
| --- | --- | --- | --- | --- | --- |
| 1 | Mitotracker Red CMXRos | Thermo Fisher Scientific | M7512 | - | 200 nM |
| 2 | vWF-FITC | Abcam | ab8822 | - | 1:500 |
| 3 | GLAST-APC | Miltenyi Biotec | 130-098-803 | ACSA-1 | 1:500 |
| 4 | CD45-FITC | Miltenyi Biotec | 130-110-637 | REA747 | 1:500 |
| 5 | CD41/61-FITC | Miltenyi Biotec | 130-124-887 | REA607 | 1:500 |

**Supplemental Table S2.** The primers used for qRT-PCR in this study.

| **Target** | **Primer sequence (5’→3’)** | **Product size (bp)** |
| --- | --- | --- |
| HIF1α-F | TATGAGCCAGAAGAACTTTTAGGC | 63 |
| HIF1α-R | CACCTCTTTTGGCAAGCATCCTG |  |
| ATG5-F | CAACTTGTTTCACGCTATATCAGG | 106 |
| ATG5-R | CACTTTGTCAGTTACCAACGTCA |  |
| BECN1 (ATG6)-F | ACCGTGTCACCATCCAGGAA | 188 |
| BECN1 (ATG6)-R | GAAGCTGTTGGCACTTTCTGT |  |
| DAPK1-F | GACCGTGAAGCATTACCTGAG | 124 |
| DAPK1-R | GCTGCTGAAGCTTTCCTTGTA |  |
| BNIP3L-F | ACAACAACAACTGC GAGGAAA | 144 |
| BNIP3L-R | GAGGATGAGGATGGTACGTGT |  |
| BAX-F | GTTTCATCCAGGATCGAGCAG | 145 |
| BAX-R | CTGCAGCTCCATGTTACTGTC |  |
| PINK1-F | GTATGAAGCCACCATGCCTAC | 153 |
| PINK1-R | CATCATCTTGATGGCCAAGGGTC |  |
| Actin-F | CATGTACGTTGCTATCCAGGC | 249 |
| Actin-R | CTCCTTAATGTC ACGCACGA |  |

**Supplemental Table S3.** List of antibodies used for Western blots in this study.

| **Target** | **Company** | **Cat No. (clone)** | **Used concentration** |
| --- | --- | --- | --- |
| HIF1α | Thermo Fisher Scientific | MA-516 (mgc3) | 1 : 1000 |
| APG5L/ATG5 | Abcam | ab108327 (EPR1755(2)) | 1 : 1000 |
| pBECN1 | Cell Signaling Technology | #84966 (D4B7R) | 1 : 1000 |
| BECN1 | Cell Signaling Technology | #3495 (D40C5) | 1 : 1000 |
| BAX | Cell Signaling Technology | #2772 | 1 : 1000 |
| Bcl-2 | Cell Signaling Technology | #4223 (D55G8) | 1 : 1000 |
| BNIP3L | Abcam | ab8399 | 1 : 1000 |
| DAPK1 | Invitrogen | PA5-14044 | 1 : 1000 |
| PINK1 | Abcam | ab23707 | 1 : 1000 |
| LC3B | Cell Signaling Technology | #3868 (D11) | 1 : 1000 |
| p62 | Santa Cruz Biotechnology | sc-48402 (A-6) | 1 : 1000 |
| Actin | Santa Cruz Biotechnology | sc-47778 (C4) | 1 : 1000 |
